# Preparation of Boron Nitride Nanotube/Aluminum Matrix Composites and Their Application in Automotive Connecting Rods

**DOI:** 10.3390/ma19010048

**Published:** 2025-12-22

**Authors:** Yong Huang, Bingzhan Zhang, Han Zhao, Qingtao Li, Jianbo Bi

**Affiliations:** School of Automotive and Traffic Engineering, Hefei University of Technology, Hefei 230009, China; huangyong563@126.com (Y.H.); hanzhao@mail.hfut.edu.cn (H.Z.); 2024171792@mail.hfut.edu.cn (Q.L.); 2024171799@mail.hfut.edu.cn (J.B.)

**Keywords:** BNNTs/Al, stirred casting method, interface optimization, automotive connecting rods

## Abstract

In order to address the urgent demand for high-performance materials in the field of automotive lightweighting, there is a need for solutions to the interface instability and performance degradation of traditional reinforcing phases (e.g., SiC, CNT) at elevated temperatures. The present study prepared BNNTs/Al composites via the stirred casting method for automotive connecting rods. The microstructure, interface characteristics, phase evolution, and high-temperature wettability were systematically characterised using a range of analytical techniques, including SEM, TEM, XRD, and DSC. A study was conducted to assess the mechanical properties of the composites in comparison to those of conventional 40Cr steel. This investigation enabled an evaluation of the material’s comprehensive performance for use in automotive connecting rods. The study successfully achieved uniform dispersion of BNNTs within the aluminium matrix, forming tightly bonded, semi-coherent interfaces such as Al/AlN and Al/AlB_2_. It was found that complete wetting was achieved at 675 °C, with interface reactions generating AlN and AlB_2_ phases that significantly enhanced performance. The prepared connecting rod demonstrates a specific strength that significantly exceeds that of 40Cr steel. The experimental investigation conducted in a controlled setting yielded notable outcomes. The empirical evidence demonstrated a 6.5% enhancement in braking performance and a 5.8% reduction in fuel consumption. Through the optimisation of interface design and process control, the BNNTs/Al composite achieves a balanced compromise between high strength, low density, and excellent thermal stability. The material’s potential for use in lightweight automotive connecting rods is significant, offering a novel approach to the eco-friendly manufacturing of related components.

## 1. Introduction

Aluminum alloys are extensively utilized in both research and development and industrial applications due to their low density, affordability, high ductility, and excellent corrosion resistance. Advancements in the industrial and technological sectors have resulted in increased demand for aluminium-based materials, which must exhibit enhanced physical, mechanical, tribological, thermal and corrosion properties to meet these demands. In comparison with aluminium alloys, aluminium matrix composites (AMCs) offer superior strength, creep resistance, wear resistance, corrosion resistance, and a lower coefficient of thermal expansion (CTE) [1,2]. It is evident that these materials demonstrate broad application potential in a variety of sectors, including aerospace [3] and automotive [4]. This is particularly relevant in the current trend towards automotive lightweighting, where these materials can effectively reduce component weight and enhance fuel economy [5,6].

However, traditional ceramic particle-reinforced aluminium matrix composites (e.g., SiC or Al_2_O_3_ reinforcements) exhibit significant degradation of mechanical properties under high-temperature conditions and insufficient bonding strength at the interface between the reinforcement and matrix, limiting their further application [7,8,9]. For instance, uncontrolled interfacial reactions at elevated temperatures readily lead to crack initiation, reducing the material’s service life. Consequently, the development of novel reinforcements to overcome these performance bottlenecks has become a major area of research in materials science [10,11].

In addition, carbon nanotubes (CNTs) have attracted significant interest in this domain. Yan et al. successfully prepared a CNT-reinforced A356 aluminium alloy casting nanocomposite using a molten metal route [12]. Zhou et al. fabricated a CNT-reinforced aluminium composite material via a pressureless infiltration process [13]. However, it has been demonstrated that CNTs are unable to withstand elevated temperatures and are susceptible to oxidation at 500 °C [14]. Furthermore, they have been observed to react readily at interfaces to form products (Al_4_C_3_), resulting in reduced wettability and affinity [15].

In contrast, boron nitride nanotubes (BNNTs) offer a superior combination of high tensile strength, large elastic modulus, exceptional thermal stability (stable up to ~900 °C in air), and strong resistance to oxidation. BNNTs share a structural resemblance to CNTs but possess a wide band gap (~5.5 eV), excellent chemical inertness, and higher flexibility, making them highly suitable for metal-matrix reinforcement in extreme-environment applications [16]. Recent reviews, such as Turhan et al. (2022), highlight BNNTs’ outstanding mechanical, thermal, and dielectric properties, as well as their growing potential in multifunctional composite systems [17].

In addition to BN-based nanostructures, aluminum nitride (AlN) nanotubes represent another important class of III–V nitride nanomaterials that exhibit high thermal conductivity, good electrical insulation, and strong covalent bonding. Their structural and electronic characteristics make AlN nanotubes relevant to metal-matrix composites and interfacial reaction studies. Moreover, the presence of defects, such as vacancies or color centers (F-centers), in BN and AlN nanotubes can significantly alter their electronic structure, chemical reactivity, and interaction with metal matrices [18]. Zhukovskii et al. (2007) reported that F-centers in AlN nanotubes induce notable variations in electronic states and bonding behavior [19], which directly influence interfacial characteristics and stabilization mechanisms in composite systems. These defect-related effects are particularly important for understanding interfacial reactions and bonding with aluminum melts.

Despite these advantages, the interfacial bonding mechanism between BNNTs and aluminum remains insufficiently understood. Current studies have not systematically clarified how BNNTs disperse, react, or form interfacial phases (such as AlN or AlB_2_) under high-temperature casting conditions. Additionally, the roles of structural defects in BNNTs and related nitride nanotubes—particularly their influence on interfacial reactivity, wettability, and phase evolution—have not been comprehensively addressed. This lack of fundamental understanding limits the optimization of processing parameters and hinders the development of BNNT-reinforced aluminum composites with stable performance.

Moreover, while BNNTs/Al composites have shown great potential in mechanical and thermal enhancement, their application in specific engineering components, such as automotive connecting rods, remains largely unexplored. Existing studies rarely extend beyond material synthesis and characterization to industrial-level component fabrication, performance validation, and practical engineering evaluation.

To address these challenges, the present study conducts an in-depth investigation into the interfacial bonding mechanism between BNNTs and the Al matrix. A systematic analysis of the interfacial characteristics of BNNTs/Al composites was conducted, thereby revealing the intrinsic relationship between interfacial bonding strength and preparation process parameters, thus elucidating the bonding mechanism. Concurrently, the preparation process for BNNTs/Al composites is being optimized. The fabrication of BNNTs/Al composite connecting rods is achieved through the implementation of a stirred casting method. The optimisation of process parameters is pivotal in ensuring the uniform dispersion of BNNTs within the Al matrix, thereby facilitating strong interfacial bonding. This, in turn, enhances the mechanical properties and operational reliability of the composite material. A comparative analysis is conducted with traditional 40Cr steel. The proposed BNNTs/Al composite connecting rod manufacturing process demonstrates promising potential for industrial translation, based on the use of conventional powder metallurgy routes and standard automotive component geometries.

## 2. Experimental Procedure

### 2.1. Preparation of BNNTs/Al Composites

The morphology of the BNNTs was characterized by SEM and TEM prior to composite fabrication. As shown in Figure S1a, the SEM image reveals a densely entangled network of fibrous nanotubes with high aspect ratios, which is typical of BNNTs. The nanotubes exhibit lengths extending to the micrometer scale. The TEM image in Figure S1b further confirms the tubular and multi-walled structure of the BNNTs, with a clearly visible hollow core. The outer diameters are mainly in the range of several tens of nanometers. No obvious amorphous phases or large particulate impurities are observed, indicating good structural purity of the BNNTs.

The composite material was achieved by precisely controlling the mass ratio of A380 aluminium powder to BNNT powder (9:1) in the intermediate preforms, with each preform set at 50 g, the resulting BNNT content in the preform was therefore 10 wt.%. The specific steps involved in this process are as follows: firstly, 5 g of BNNT powder must be dispersed uniformly in 50 millilitres of anhydrous ethanol, and then the mixture is subjected to ultrasonic treatment for a duration of 60 min using an ultrasonicator, with the objective of ensuring thorough dispersion. Subsequently, 45 g of 200-mesh A380 aluminum powder is added to the dispersion, and the two components are thoroughly mixed using a magnetic stirrer. The mixture is then placed in a vacuum drying oven and dried at 60 °C to remove ethanol. The dried powder is then subjected to further grinding in a planetary ball mill operating (the ball-to-powder ratio (BPR) was 15:1) at a speed of 150 revolutions per minute in for a duration of two hours. Milling was conducted under nitrogen atmosphere to prevent oxidation. During this process, stearic acid (1.0 wt.%) is incorporated into the milling media with the objective of averting the occurrence of powder cold welding. The mixture was then compressed into the desired BNNTs/Al intermediate preform using a dry powder hydraulic press at 200 MPa pressure, following which the preform was held for a period of five minutes. The BNNT preform was fabricated in a cylindrical geometry, with dimensions on the millimeter scale. The cylindrical preform was vertically introduced into the molten aluminum for the infiltration process.

The specific process for preparing BNNTs/Al intermediate preforms based on the aforementioned preforms is as follows. The fusion process was carried out under ambient atmospheric conditions without the use of any protective gas. The BNNTs were first fabricated into a preform before being introduced into molten aluminum, rather than being added directly as loose powders. This approach was adopted to prevent severe agglomeration and floating of BNNTs caused by their low density and poor wettability with molten aluminum, and to facilitate a more uniform infiltration and distribution of BNNTs within the aluminum matrix.

In the initial phase of the process, a precise quantity of A380 aluminium alloy is subjected to heating to a temperature of 780 °C within an electric resistance furnace, thereby achieving the desired state of melting. Subsequently, the intermediate preforms being added to the melt in small batches. Concurrently with preform addition, high-energy ultrasonic treatment is applied to the melt using an ultrasonic-assisted agitation device at a frequency of 20 kHz. Ultrasonic treatment was performed using a probe-type ultrasonic device at a power of 700 W for 15 min. The ultrasonic probe was inserted approximately 20–30 mm below the melt surface to generate strong cavitation and facilitate the breakup of BNNT agglomerates. Subsequent to ultrasonic treatment, dross removal is conducted to eliminate impurities from the melt. The melt was subsequently cast into steel molds. Following a thorough consolidation and cooling process, the targeted BNNTs/Al composite aluminium matrix composite was successfully obtained. Moreover, BNNTs/Al aluminium matrix composites were successfully prepared using this method for comparative analysis.

### 2.2. Preparation of BNNTs/Al Composite Materials for Automotive Connecting Rods

The present study employed the stirred casting method to fabricate BNNTs/Al composite connecting rods. Stir casting was carried out at a stirring speed of 500 rpm for 10 min to promote uniform dispersion of BNNTs in the melt. After mechanical stirring, the melt was held at 760 °C for 10 min before casting. The steel mold was preheated to 200 °C to reduce thermal shock and improve filling quality. Through the optimisation of process parameters, the uniform dispersion of BNNTs within the Al matrix was achieved, resulting in strong interfacial bonding. Consequently, the mechanical properties and operational reliability of the composite material were enhanced. The manufacturing process of automotive connecting rods, as demonstrated in Text S1 and Figure S2, is composed of four primary stages: dimension determination, mold machining, connecting rod blank fabrication, and connecting rod finishing.

### 2.3. Structural Characterization of BNNTs/Al Composites

First, the microstructure of the composite material was observed and analyzed using a high-resolution scanning electron microscope (SEM, Tescan-Vega3, Shanghai, China) combined with energy dispersive spectroscopy (EDS, TESCAN CHINA, Shanghai, China). The application of EDS enabled quantitative analysis of elemental distribution within the composite, with particular focus on the interface interactions between boron and nitrogen elements in BNNTs and the aluminum matrix elements. This provided intuitive and scientific evidence for elucidating the reinforcement mechanism of the composite. Subsequently, to investigate the fine structure of the composite at the nanoscale, transmission electron microscopy (TEM, Talos F200X, Thermo Fisher Scientific, Hillsboro, OR, USA) was employed for analysis. Additionally, to accurately analyze the phase composition and crystal structure of the composite material, X-ray diffraction analysis (XRD, D8 ADVANCE, Bruker AXS GmbH, Karlsruhe, Germany) was employed. Finally, to evaluate potential structural damage to BNNTs during composite preparation and assess the impact on nanotube structural stability, Raman spectroscopy analysis (alpha300R, WITec GmbH, Ulm, Germany) was utilized.

### 2.4. Contact Angle and Mechanical Testing

This experiment comprehensively evaluated the wetting properties of the BNNTs/Al composite system under different temperature conditions, along with its mechanical properties at room temperature and elevated temperatures. The study aimed to gain insights into droplet morphology at each temperature point, measure and calculate contact angles on both sides of the droplet, and characterize mechanical response properties under varying environmental conditions. The contact angle was measured using the sessile drop method. For each temperature, three independent measurements were performed, and the contact angles were obtained using ImageJ (version 1.53t, National Institutes of Health, Bethesda, MD, USA)with the DropSnake plugin. All wettability experiments were performed using a high-temperature, high-vacuum contact angle analyzer under a vacuum level better than 1 × 10^−5^ Pa. Detailed procedures are outlined in Texts S2 and S3, and illustrated in Figures S3 and S4.

## 3. Results and Discussion

### 3.1. BNNTs/Al Composite Microstructure

**Figure 1:**
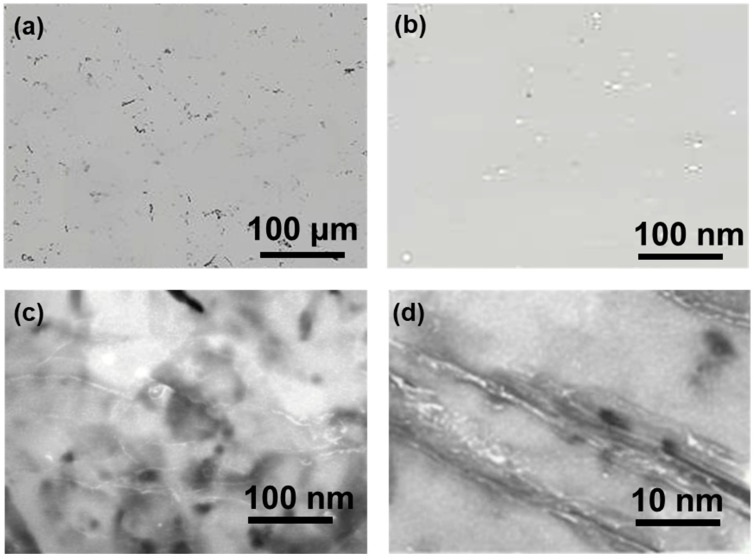
BNNTs/Al composite materials: (**a**,**b**) SEM, (**c**,**d**) TEM microstructure.

As illustrated in Figure 1, the microstructure of the BNNTs/Al composite material was prepared via the stirred casting method. The low-magnification SEM image in Figure 1a reveals a uniform matrix structure with no visible defects, such as bubbles or impurities. This finding suggests that the process parameters used for the stirring casting stage were optimized and controlled effectively, thereby preventing the introduction of additional defects during the composite preparation stage and ensuring the high quality of the material. The uniform distribution of fine black dots within the matrix is indicative of the excellent dispersion state of the BNNTs. In order to facilitate further analysis of the dispersion of BNNTs in the matrix, Figure 1b presents a local magnification of Figure 1a. The image clearly demonstrates that BNNTs are uniformly dispersed in the Al matrix as single or small clusters of fibres, with no noticeable agglomeration observed. The nanoscale dimensions and high aspect ratio of the BNNTs indicate that their structural integrity was well preserved during composite fabrication.

Figure 1c,d illustrate the transmission electron microscopy (TEM) microstructure of the BNNTs/Al composite, which was prepared via the stirred casting method. The low-magnification TEM image in Figure 1c reveals a uniform matrix structure, with white BNNTs exhibiting uniform dispersion and no noticeable agglomeration. This finding suggests that the stirred casting process effectively promotes the dispersion of BNNTs in the Al melt, thereby forming an ideal composite microstructure. The uniform distribution of BNNTs within the matrix provides a crucial microstructural foundation for their reinforcement and toughening effects. In order to facilitate further analysis of the interface bonding between BNNTs and the Al matrix, Figure 1d presents a higher-magnification TEM image. The image clearly reveals a tightly bonded interface between BNNTs and the Al matrix, with no discernible defects such as bubbles or impurities. The superior quality of the interface bonding can be attributed primarily to the exceptional surface wettability exhibited by BNNTs and their seamless interface compatibility with aluminium. The absence of a chemically inert oxide layer on BNNT surfaces facilitates thorough interface reactions with the Al melt, resulting in a robust interface bond. This tight interface bonding effectively transmits stress, thereby maximising the reinforcing effect of BNNTs while simultaneously inhibiting crack initiation and propagation at the interface. This process enhances the toughness of the composite material. The microstructural characteristics of uniformly dispersed BNNTs tightly bonded to the Al matrix are crucial for the mechanical properties of the composite. A substantial corpus of research has demonstrated that when BNNTs are uniformly dispersed as reinforcements in the Al matrix, the composite exhibits significant improvements in strength, hardness, and toughness. For instance, Singhal et al. observed that incorporating BNNTs increased the yield strength and fracture elongation of Al-based composites by over 50–80% [17]. This enhancement is primarily attributed to the uniform dispersion of BNNTs and their efficient load transfer mechanism.

### 3.2. Evolution Mechanism of Nanotube/Aluminum Microstructures

#### 3.2.1. Characteristics of the BNNTs/Al Interface

**Figure 2:**
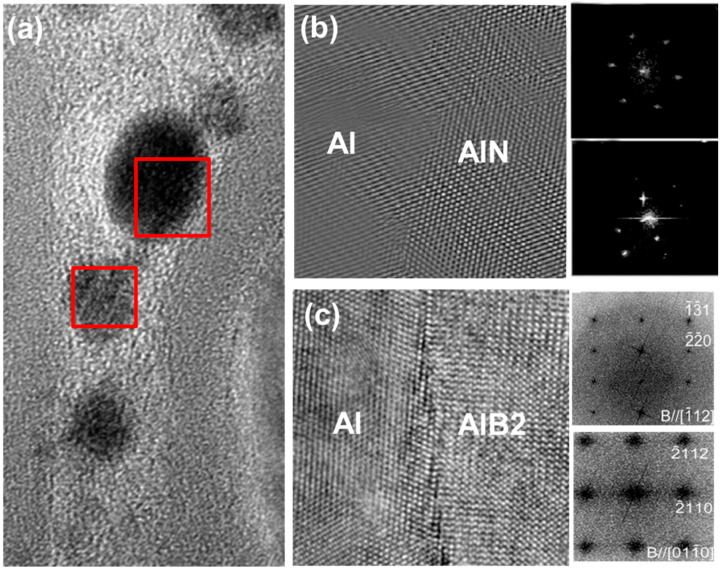
(**a**) The red box highlights the interface characteristics of BNNTs/Al.; (**b**) Al/AlN semi-coherent interface characteristics; (**c**) Al/AlB_2_ semi-coherent interface characteristics.

As illustrated in Figure 2, the interface characteristics between BNNTs and the Al matrix in BNNTs/Al composites are of particular interest. The interface structure and properties have been shown to significantly influence the mechanical and thermal conductivity properties of the composite material. A comprehensive investigation was undertaken to ascertain the interface characteristics of BNNTs/Al composites from a crystallographic perspective, with a view to ascertaining their potential influence on composite performance. Figure 2a presents a high-resolution transmission electron microscopy (HRTEM) image of the BNNTs/Al composite, clearly revealing the interface structure between BNNTs and the Al matrix. The image displays BNNTs exhibiting a typical multi-walled nanotube structure, with tube walls oriented parallel to the interface direction. A tightly bonded interface has been observed to form between BNNTs and the Al matrix, with no significant interface defects or voids identified. The superior quality of the interface bonding can be attributed to the optimal lattice matching and chemical compatibility between BNNTs and Al.

Figure 2b illustrates the characteristics of the semi-coherent interface between the Al(111) plane and the AlN(0002) plane. According to crystallographic analysis, the interplanar spacing of the Al(111) plane is 0.2338 nm, while that of the AlN(0002) plane is 0.2490 nm [20]. A 7% lattice mismatch exists between the two crystal planes, indicating the formation of a semi-coherent interface between Al and AlN. At the interface, Al atoms form stable chemical bonds with N atoms, while adjacent Al atoms exhibit weak van der Waals interactions with B atoms on the BNNT wall. This alternating arrangement of chemical bonding and physical adsorption enhances the interface’s bonding strength and stability. Figure 2c illustrates the characteristics of the semi-coherent interface between the Al(111) plane and the AlB_2_(0001) plane. AlB_2_ is a typical hexagonal compound whose (0001) plane also exhibits a degree of lattice matching with the Al(111) plane. The interplanar spacing of the AlB_2_(0001) plane is 0.3009 nm, exhibiting a 22% lattice mismatch with the Al(111) plane (0.2338 nm)—higher than that observed at the Al/AlN interface [21,22]. Nevertheless, a semi-coherent interface can still form between Al and AlB_2_. At the interface, Al atoms form strong covalent bonds with B atoms, while adjacent Al atoms form weak van der Waals forces with N atoms on the BNNT walls. This alternating interface bonding pattern, similar to the Al/AlN interface, enhances the interface bonding strength and stability. The presence of semi-coherent interfaces in BNNTs/Al composites significantly impacts both mechanical and thermal properties. First, these interfaces enhance bonding strength between BNNTs and the Al matrix. Robust interface bonding enables efficient stress transfer, leveraging BNNTs’ reinforcing effect to boost composite strength and modulus [23]. When the composite is subjected to external forces, stresses can be efficiently transferred from the matrix to the BNNTs via the interface. The BNNTs can then bear substantial loads without interfacial debonding or slip. This stress transfer mechanism allows BNNTs to fully leverage their superior mechanical properties, significantly enhancing the composite’s strength and toughness.

Secondly, the semi-coherent interface has the capacity to influence the thermal conductivity of the composite material. It is evident that BNNTs themselves possess extremely high thermal conductivity; however, within the composite, their thermal performance is largely contingent on the interfacial thermal resistance with the matrix. The presence of a semi-coherent interface has been demonstrated to assist in the reduction of interfacial thermal resistance, thereby facilitating phonon transmission at the interface [24]. As demonstrated by crystallographic analysis, the semi-coherent interfaces formed between aluminium (Al) and aluminium nitride (AlN) or aluminium boride (AlB_2_) exhibit analogous lattice vibration characteristics. This facilitates phonon scattering and transmission at the interface. In comparison with non-congruent interfaces, semi-coherent interfaces offer a greater number of phonon transmission pathways, thereby reducing energy dissipation caused by phonon scattering at the interface [25].

The characteristic E_2_g Raman mode of BNNTs appears near 1367 cm^−1^, with only a slight intensity reduction and negligible peak shift after aluminium melt processing, indicating that the tubular structure of BNNTs remains intact. A weak low-frequency RBM-like signal is also observed near~52 cm^−1^, consistent with multi-walled BNNTs. The similarity of the two spectra confirms that no significant structural degradation occurred during composite fabrication (Figure S5).

Quantitative measurement of the thickness of the AlN and AlB_2_ interfacial layers was not included in the present work because the primary objective of the TEM analysis was to reveal the structural characteristics and bonding state at the nanotube/Al interface. Although the interfacial reaction products are clearly observed in the HRTEM images, their thickness varies at different locations and does not form a uniform continuous layer. Future work will include statistical measurement of interface thickness and EDS line-scan analysis to provide a more comprehensive quantification of the interfacial reaction zone. While SEM and TEM observations confirm that BNNTs are uniformly dispersed in the aluminium matrix without significant agglomeration, a quantitative statistical analysis such as cluster size distribution was not performed in this study. The present work focuses primarily on establishing the feasibility of BNNT dispersion via the stirred casting method. A quantitative dispersion analysis based on image segmentation and particle size statistics will be conducted in future work to more precisely evaluate the distribution uniformity of BNNTs.

In addition to load transfer, the presence of well-bonded and uniformly dispersed BNNTs also acts as effective obstacles to dislocation motion in the aluminum matrix. Dislocation pinning and bowing around BNNTs and interfacial reaction phases increase the resistance to plastic deformation, thereby contributing to strength enhancement while maintaining ductility.

#### 3.2.2. Thermogravimetric Analysis of BNNTs/Al

**Figure 3:**
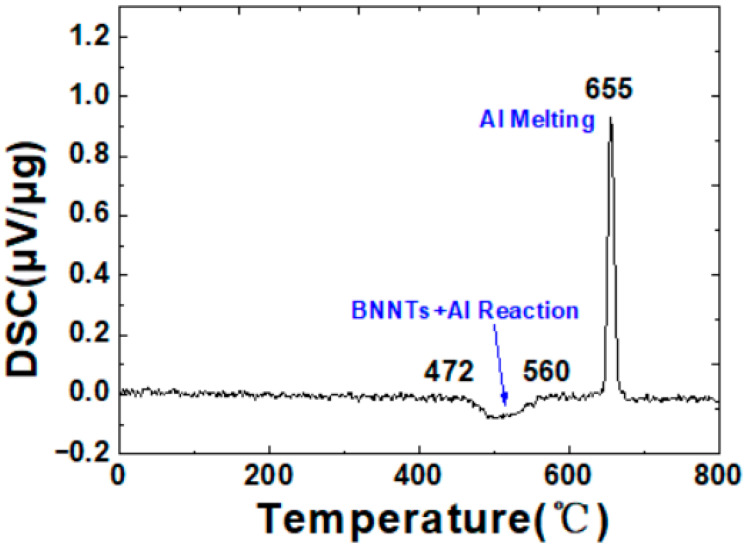
Analysis of DSC Curves for BNNTs/Al Composites.

The present study employed differential scanning calorimetry (DSC) to characterise the thermal behaviour of BNNTs/Al composites, with a view to investigating the interfacial reaction mechanism of the composites. In order to further explore the interfacial reaction mechanism of BNNTs/Al composites, Figure 3 displays the DSC curve of the BNNTs/Al composite during the heating process. The figure reveals a distinct exothermic peak in the DSC curve between 472 °C and 560 °C. This peak is consistent with the interfacial reaction between BNNTs and Al. Within this temperature range, nitrogen atoms on the BNNT surface undergo chemical reactions with the aluminium matrix, forming an AlN interfacial phase. The formation of the AlN interfacial phase has been demonstrated to enhance the interface bonding between BNNTs and Al, thereby contributing to improved mechanical properties and thermal stability of the composite. As the temperature continues to rise, a further endothermic peak becomes observable at approximately 655 °C. This peak is indicative of the melting of the Al matrix. Following the melting process, liquid Al demonstrates enhanced wetting and encapsulation properties with regard to BNNTs, thereby facilitating their dispersion within the Al matrix. Concurrently, the process of aluminium melting generates pathways for atomic migration, thereby facilitating subsequent BNNT-Al reactions and accelerating interfacial interactions [25]. Following the melting process, the reactions between BNNT-Al continue until thermodynamic equilibrium is attained. The temperature range and reaction mechanisms governing BNNT/Al composite interfacial reactions have been elucidated through DSC-TGA analysis.

#### 3.2.3. Evolution of BNNTs/Al High-Temperature Wettability

**Figure 4:**
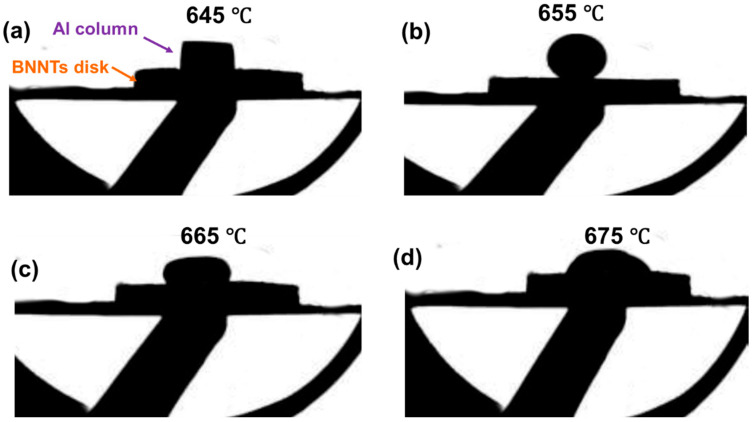
Wetting angle diagrams of BNNTs/Al at different temperatures: (**a**) 645 °C, (**b**) 655 °C, (**c**) 665 °C, (**d**) 675 °C.

Boron nanotubes (BNNTs) are regarded as optimal materials for enhancing the performance of aluminium-based composites, owing to their exceptional mechanical properties, thermal conductivity and chemical stability. Nevertheless, the wettability and interfacial bonding strength between BNNTs and the aluminium matrix are critical factors affecting composite performance. The present study employs contact angle measurement to systematically investigate the wetting behaviour of the BNNTs/Al system at different temperatures. The objective of this study is to elucidate the impact of temperature on the wettability of BNNTs/Al, with the aim of providing theoretical guidance to optimise the preparation process of BNNTs/Al composites. Figure 4 presents the contact angle images of the BNNTs/Al system at varying temperatures. The figures demonstrate that temperature exerts a significant influence on the wetting behaviour of the BNNTs/Al system. At a temperature of 645 °C (see Figure 4a), the aluminium block demonstrates negligible deformation and a high contact angle, thus indicating poor wetting between aluminium and BNNTs. This is primarily because at 645 °C, the aluminium block remains in a solid state with limited atomic mobility, hindering its ability to spread across the BNNT surface and resulting in poor wetting. Concurrently, the van der Waals forces between aluminium and BNNTs are found to be weak, thereby impeding the process of overcoming aluminium’s surface tension and the establishment of stable interfacial bonding. As the temperature increases to 655 °C (Figure 4b), the aluminium block begins to melt, forming droplets. Despite its liquid state, aluminium exhibits only partial spreading across the BNNT surface, manifesting a substantial contact angle and inadequate wettability. The aetiology of this phenomenon may be multifaceted, involving a combination of factors. Firstly, the low surface energy of BNNTs creates significant interfacial tension with aluminium droplets, thereby hindering spreading. Secondly, adsorbed impurities or oxide layers on BNNT surfaces have the potential to disrupt chemical reactions and interfacial bonding with aluminium droplets [26,27]. Thirdly, the fluidity and wettability of the aluminium droplet at 665 °C (Figure 4c) may be inadequate to fully cover the BNNT surface and form a continuous coating layer.

As the temperature was increased to 675 °C (Figure 4d), a significant enhancement in the wettability of the BNNTs/Al system was observed. The aluminium droplets are distributed across the surface of the BNNTs, thus establishing a continuous coating layer. The contact angle decreased to 0°, achieving complete wetting. The following factors have been identified as the primary contributors to this phenomenon: Firstly, the enhanced flowability and wettability of the aluminium droplets at elevated temperatures facilitated their spreading on the BNNT surface. Secondly, elevated temperatures promoted interfacial reactions between the aluminium droplets and BNNTs, generating interfacial phases such as AlN and AlB_2_, which enhanced interfacial bonding strength. Thirdly, elevated temperatures have been shown to reduce the surface tension of aluminium droplets, thereby decreasing the resistance to spreading on the BNNT surface. The complete wetting of BNNTs by molten aluminium droplets facilitates uniform and continuous coating, thereby enhancing the density and mechanical properties of the composite material [28].

#### 3.2.4. Phase Evolution of BNNTs/Al at High Temperatures

**Figure 5:**
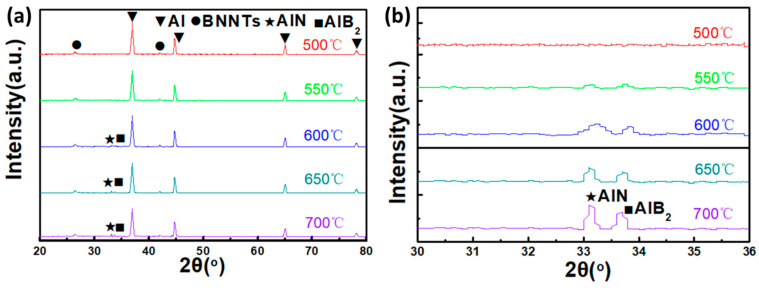
(**a**) Phase structure of BNNTs/Al at different temperatures, (**b**) is a local magnification of (**a**).

The interfacial reactions and phase evolution between BNNTs and Al exert a decisive influence on the properties of the composite material. The present study employs X-ray diffraction (XRD) to systematically investigate the phase evolution behaviour of BNNTs/Al composites within the 500–700 °C temperature range. The diffraction data reflect the phase constitution after high-temperature exposure rather than in situ diffraction from the molten aluminum. The objective of this study is to elucidate the reaction mechanism and phase evolution patterns between BNNTs and Al at elevated temperatures, with a view to providing theoretical guidance for the optimisation of the preparation process and performance regulation of BNNTs/Al composites. Figure 5 presents the XRD patterns of BNNTs/Al composites at varying temperatures. As demonstrated in Figure 5a, at 500 °C, the composite predominantly comprises Al and BNNTs, with no substantial new phase formation, suggesting that no notable chemical reaction occurs between BNNTs and Al. The following reasons have been identified as the primary causes of this phenomenon: Firstly, it is evident that the 500 °C temperature is inadequate in providing sufficient energy to facilitate interfacial reactions between BNNTs and Al. Secondly, the presence of adsorbed impurities or an oxide layer on the BNNT surface has the potential to impede direct contact and reaction with Al. Thirdly, Al remains in a solid state at 500 °C, exhibiting limited atomic mobility, thereby rendering effective interfacial reactions with BNNTs challenging. As the temperature rises above 550 °C, the phase composition of the BNNTs/Al composite begins to undergo significant changes.

As demonstrated in the magnified segment of Figure 5b, the distinctive diffraction peaks of the AlN and AlB_2_ phases emerge in a gradual manner within the temperature range of 550–700 °C, with peak intensities undergoing a progressive enhancement with increasing temperature. This finding signifies an interfacial reaction between BNNTs and Al, resulting in the formation of AlN and AlB_2_ reaction products. It is noteworthy that the reaction intensity exhibits an increase with rising temperature. The formation mechanisms of AlN and AlB_2_ are outlined as follows: Initially, at elevated temperatures, the B-N bonds on the BNNT surface are broken, releasing active B and N atoms. Concurrently, the aluminium atoms attain sufficient energy to become reactive and mobile. Subsequently, the free B and N atoms undergo a chemical reaction with Al atoms, forming AlN and AlB2, respectively [23,29].

BN(s) + Al(s) → AlN(s)(1)

2BN(s) → 2B(s) + N_2_(g) (2)

2B(s) + 2Al(s) → AlB_2_(s) (3)

The phase evolution behaviour of BNNTs/Al composites at elevated temperatures is a key factor influencing their mechanical properties, thermal conductivity, and service stability. It has been established through prior research that interfacial reactions between BNNTs and Al occur at temperatures in excess of 550 °C, resulting in the formation of AlN and AlB_2_ reaction products. Furthermore, it has been demonstrated that the intensity of these reactions increases in proportion to the increase in temperature. However, it should be noted that temperature is not the sole factor influencing phase evolution in BNNTs/Al composites; holding time also significantly affects the interfacial reaction process and product composition. The present study employs X-ray diffraction (XRD) to systematically investigate the phase evolution behaviour of BNNTs/Al composites during 25–40 min heat treatment at 700 °C. The objective of this study is threefold: firstly, to reveal the influence of holding time on BNNTs/Al interface reactions; secondly, to deepen understanding of the high-temperature reaction mechanism in BNNTs/Al composites; and thirdly, to provide theoretical guidance for process optimization and performance regulation.

**Figure 6:**
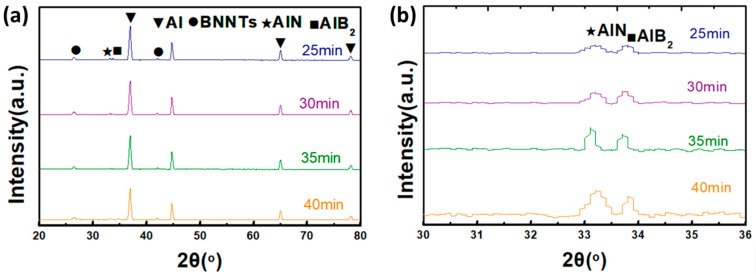
(**a**) Phase evolution of BNNTs/Al over time, (**b**) is a local magnification of (**a**).

As illustrated in Figure 6, the XRD patterns of BNNTs/Al composites vary according to the duration of exposure to 700 °C. As demonstrated in Figure 6a, the phase composition of the composite undergoes substantial alterations as the holding time increases from 25 min to 40 min. During the initial holding period (25 min), the intensities of the characteristic peaks for AlN and AlB_2_ were relatively weak, indicating limited formation of interfacial reaction products. This phenomenon can be attributed primarily to the short reaction time, during which atomic diffusion and chemical reactions between BNNTs and Al had not yet occurred, thereby restricting the nucleation and growth of reaction products [30]. As the holding time was extended to 30 min and 35 min, the intensities of the AlN and AlB_2_ characteristic peaks gradually increased, indicating the ongoing interfacial reaction and continuous accumulation of reaction products. As the holding time increased to 40 min, the intensities of the AlN and AlB_2_ characteristic peaks increased further, indicating a substantial intensification of the interfacial reaction and the generation of a significant quantity of reaction products. The evolution trend of AlN and AlB_2_ characteristic peaks with holding time can be observed more clearly in the local magnification of Figure 6b. As the holding time was increased from 25 min to 40 min, both AlN and AlB2 diffraction peak intensities exhibited a gradual increase. This finding serves to further corroborate the hypothesis that prolonging the holding time is conducive to enhancing the interfacial reaction between BNNTs and Al, thereby promoting the formation of AlN and AlB_2_ reaction products. Although aluminum oxidation is thermodynamically possible at elevated temperatures, the low-oxygen processing conditions employed in this study effectively suppressed oxidation, and thus the interfacial evolution is dominated by nitridation and boridation reactions.

In consideration of the preceding research into the reaction mechanisms at the BNNTs/Al composite interface, the phase evolution behaviour under the influence of holding time can be explained as follows: Initially, at an elevated temperature of 700 °C, BNNTs and Al atoms obtain sufficient energy to break their original bonds, thereby becoming highly reactive and mobile. As the holding time increases, the diffusion distance of BNNTs and Al atoms is extended, thereby enhancing collision and reaction probabilities and thus promoting interfacial reactions [25]. Secondly, the prolongation of the holding time facilitates a more thorough atomic rearrangement and structural adjustment at the reaction interface. This has been shown to enhance the kinetic conditions for AlN and AlB_2_ nucleation and growth, consequently leading to increased crystallinity and yield of reaction products. Thirdly, the accumulation of AlN and AlB_2_ reaction products alters the composition and structure of the reaction interface, thereby influencing subsequent reactions. As the holding time increases, AlN and AlB_2_ form continuous, dense transition layers at the interface. These layers impede direct contact between BNNTs and Al atoms, while concomitantly establishing novel pathways for interface reactions, thereby fostering sustained reaction progression [31]. It is important to note that while extended holding times have been shown to enhance AlN and AlB_2_ formation, excessively long durations may also be detrimental. Excessive interfacial reactions have been shown to result in structural damage and defect generation in BNNTs, thereby weakening their reinforcing effect on the matrix [32]. Furthermore, excessive AlN and AlB_2_ formation has been shown to significantly alter the composite’s composition and microstructure, adversely affecting its mechanical properties and service stability [33]. Consequently, during the fabrication of BNNTs/Al composite, it is imperative to comprehensively evaluate the combined effects of temperature and holding time, optimise process parameters, and control the interfacial reaction process to achieve an optimal balance of composite properties.

From the perspective of composite design, the formation of secondary phases such as AlN and AlB_2_ is not inherently detrimental. A limited amount of these interfacial reaction products can improve interfacial bonding between BNNTs and the aluminum matrix, thereby facilitating effective load transfer. However, excessive formation of brittle AlN and AlB_2_ phases resulting from prolonged exposure or overly aggressive processing conditions may deteriorate interfacial integrity and negatively affect the mechanical performance of the composite. Therefore, controlling the extent of interfacial reactions is critical to achieving a balance between interfacial bonding and phase stability in BNNT-reinforced aluminum composites.

## 4. Application of BNNTs/Al on Automotive Connecting Rods

**Figure 7:**
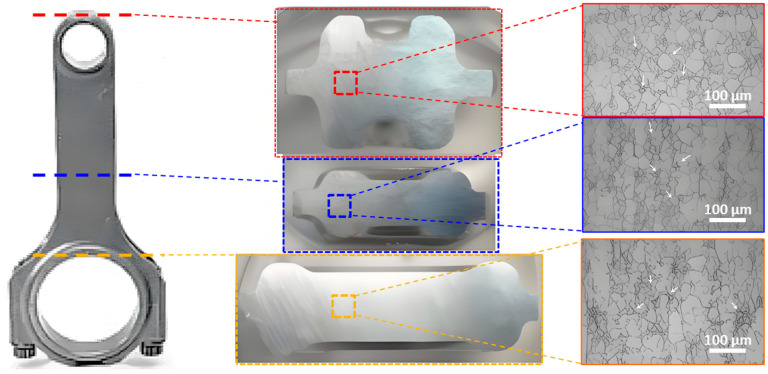
SEM microstructures of BNNTs/Al composite at different locations of the automotive connecting rod. The BNNTs dispersed in the Al matrix are highlighted by arrows. Red, green, and yellow indicate the small end, connecting rod body, and big end respectively.

BNNTs/Al composites have been demonstrated to have broad application prospects in the lightweight design and manufacturing of automotive connecting rods. This is due to their outstanding mechanical properties, thermal conductivity and vibration damping capabilities. The present study aims to evaluate the microstructure and mechanical properties of BNNTs/Al composite connecting rods. In order to achieve this objective, the study systematically characterises different regions of the connecting rod using scanning electron microscopy (SEM), transmission electron microscopy (TEM), and mechanical testing. The focal point of this study pertained to the dispersion state of BNNTs within the Al matrix, the interface bonding conditions, and their impact on connecting rod performance. The results indicate that an optimized stirred casting process can produce BNNTs/Al composite connecting rods with uniform microstructure and excellent mechanical properties, laying a solid foundation for their widespread application in the automotive industry. As illustrated in Figure 7, the microstructural morphology of the BNNTs/Al composite connecting rod varies according to the location. A preliminary investigation into the matrix structure of the three representative regions (big end, small end, and web) was conducted using low-magnification SEM images. As indicated by the arrows in Figure 7, the BNNTs appear as fine fibrous features uniformly embedded in the aluminum matrix at all examined locations. The results indicate that the matrix structure is uniformly dense, with no apparent casting defects such as shrinkage porosity or shrinkage cavities. This finding suggests that the stirred casting process effectively regulates the solidification process and suppresses defect formation. It can be observed that BNNTs are uniformly dispersed throughout the Al matrix in all three regions—big end, small end, and web—with no significant agglomeration or precipitation phenomena. This uniformity is attributed to the shear forces and turbulent effects during the stirred casting process, which ensure thorough dispersion and mixing of BNNTs within the Al melt [34]. Moreover, no significant interfacial voids or debonding were observed between BNNTs and the Al matrix, indicating excellent interfacial bonding between the two.

**Figure 8:**
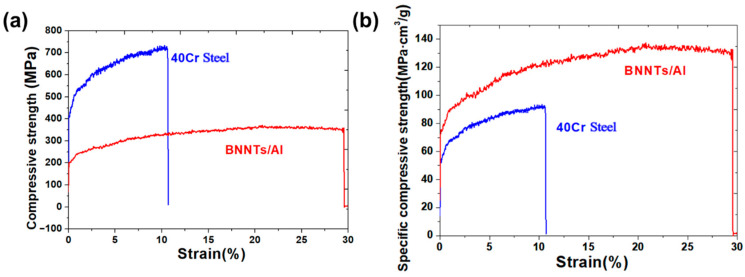
Comparison Between BNNTs/Al Composite Material for Automotive Connecting Rods and Conventional 40Cr Steel (**a**) Comparison of compression properties; (**b**) Comparison of specific strength and compression properties.

The mechanical properties of the material in automotive connecting rod applications were the focus of a study that simultaneously evaluated and compared them with those of conventional 40Cr steel. As illustrated in Figure 8, a comparison is presented of performance test results under compressive loading for the BNNTs/Al composite and 40Cr steel. As demonstrated in Figure 8a, the compressive strength of 40Cr steel is considerably higher than that of the BNNTs/Al composite. The compressive yield strength of 40Cr steel is approximately 717 MPa, while that of the BNNTs/Al composite is about 385 MPa, with the former being approximately 1.86 times that of the latter. This is primarily due to the quenching and tempering treatment of 40Cr steel, which results in the formation of a mixed microstructure comprising martensite and bainite. This process leads to the development of high strength and hardness [35]. Despite the presence of high-strength BNNTs within the BNNTs/Al composite, the matrix of the composite consists of a soft aluminium alloy, thereby resulting in an overall strength level that is comparatively low. However, the shape of the compression deformation curve indicates that the BNNTs/Al composite exhibits superior plastic deformation capability. Upon attaining its yield strength, the BNNTs/Al composite exhibits a capacity to withstand substantial compressive strain without a discernible decline in stress, thereby demonstrating remarkable strain hardening effects and ductility. Conversely, 40Cr steel demonstrates a swift decline in stress with rising strain post-yield strength, signifying comparatively diminished plastic deformation capacity and heightened vulnerability to brittle fracture.

Figure 8a also presents the elongation data of both materials under compressive loading. The compressive elongation of the BNNTs/Al composite was found to be approximately 29.4%, which is significantly higher than the 11.2% observed for 40Cr steel. This finding suggests that the BNNTs/Al composite possesses an enhanced resistance to compressive deformation without fracture, demonstrating superior plasticity and toughness. The primary factor contributing to this performance is the exceptional mechanical properties of BNNTs, which exhibit a high interfacial bonding strength. BNNTs are uniformly dispersed within the aluminium matrix, forming a dense nanoscale network structure that effectively hinders dislocation movement and propagation. This, in turn, enhances the composite’s strength and toughness [36,37]. A robust interfacial bond between BNNTs and the aluminium matrix efficiently transfers loads and mitigates stress concentration, improving the composite’s deformation capacity and fracture toughness. Beyond compressive strength and elongation, material density exerts a substantial influence on the practical applications of the material. The density of 40Cr steel is approximately 7.85 g/cm^3^, while the BNNTs/Al composite measures about 2.7 g/cm^3^—the former being roughly 2.9 times denser than the latter. This indicates that for equivalent volumes, the density of 40Cr steel (7.85 g/cm^3^) is approximately 2.9 times that of the BNNTs/Al composite (2.7 g/cm^3^). Consequently, when assessing a material’s mechanical properties, it is imperative to introduce the concept of specific strength, defined as the ratio of a material’s strength to its density.

Figure 8b presents a comparison of the compressive strength-to-density ratio for BNNTs/Al composites and 40Cr steel. The compressive strength-to-density ratio of 40Cr steel is approximately 91.3 MPa·cm^3^/g, while that of BNNTs/Al composites is about 142.6 MPa·cm^3^/g, representing approximately 1.56 times that of the former. This finding suggests that, despite the composite’s lower absolute strength compared to 40Cr steel, its specific strength performance exceeds that of 40Cr steel when density is taken into account. This advantage is primarily attributable to the superior specific stiffness and specific strength of the BNNTs/Al composite. BNNTs possess inherently elevated strength and modulus, while maintaining a density comparable to carbon nanotubes. When dispersed uniformly in the aluminium matrix at a low volume fraction, BNNTs have been shown to significantly enhance the composite’s mechanical properties while preserving low density. This property endows BNNTs/Al composites with a distinct advantage in specific strength.

**Figure 9:**
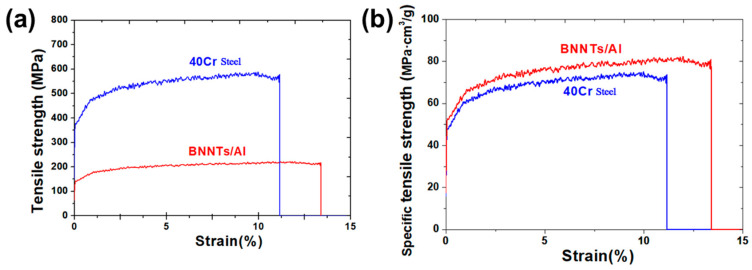
Comparison of Tensile Properties Between BNNTs/Al Composite Connecting Rods and Conventional 40Cr Steel (**a**) Tensile property comparison; (**b**) Specific strength tensile property comparison.

The tensile properties of the material in automotive connecting rod applications were the subject of further evaluation and comparison with conventional 40Cr steel. As illustrated in Figure 9, a comparison is presented of performance test results under tensile loading for the BNNTs/Al composite and 40Cr steel. As demonstrated in Figure 9a, the tensile strength of 40Cr steel is considerably higher than that of the BNNTs/Al composite, as evidenced by the tensile performance curve. The tensile yield strength of 40Cr steel is approximately 590 MPa, while that of the BNNTs/Al composite is about 212 MPa, with the former being approximately 1.36 times that of the latter. The fundamental reason for this discrepancy is attributable to the intrinsic composition and structural characteristics of the materials. Following quenching and tempering, 40Cr steel develops a composite microstructure of tempered martensite and carbides, thereby acquiring high strength and hardness. Despite the presence of high-strength BNNTs within the BNNTs/Al composite, the matrix of the composite is a soft aluminium alloy, thereby resulting in an overall strength level that is comparatively low. Despite the BNNTs/Al composite’s lower tensile strength in comparison to 40Cr steel, the shape of its tensile deformation curve indicates that it exhibits plastic deformation capabilities that are comparable to those of 40Cr steel. The tensile elongation data for both materials is comparable, with 40Cr steel exhibiting an approximate value of 11.2% and BNNTs/Al composites demonstrating a similar figure of around 11.4%. This finding suggests that BNNTs/Al composites possess the capacity to undergo substantial plastic deformation under tensile loading conditions without succumbing to premature fracture, thereby demonstrating exceptional ductility and toughness. This phenomenon is primarily attributed to the uniform dispersion of BNNTs within the aluminium matrix and their effective load transfer function. A robust interfacial bond is formed between BNNTs and the aluminium matrix, which effectively suppresses dislocation movement and impedes crack propagation, thereby enhancing the composite’s plastic toughness [38]. In addition to strength and ductility, the elastic modulus is an important parameter reflecting the stiffness of structural materials. The elastic modulus was experimentally determined from the initial linear region (strain < 0.2%) of the tensile stress–strain curves by linear fitting. The Young’s modulus of the BNNTs/Al composite is approximately 70 GPa (E = 105/0.0015 = 70 GPa), whereas that of 40Cr steel is about 213 GPa (E = 320/0.0015 = 213 GPa). This difference is mainly attributed to the intrinsic modulus of the aluminum matrix compared with steel.

In addition to tensile strength and elongation, the specific strength of a material is a crucial indicator of its lightweight performance. Figure 9b presents a comparison of the tensile specific strength of BNNTs/Al composites and 40Cr steel. The material under consideration has a density of approximately 7.85 g/cm^3^ and a tensile yield strength of about 590 MPa. This results in a tensile specific strength of approximately 75.2 MPa·cm^3^/g. The BNNTs/Al composite has a density of approximately 2.7 g/cm^3^ and a tensile yield strength of about 212 MPa, resulting in a tensile specific strength of approximately 78.5 MPa·cm^3^/g. It has been demonstrated that, despite the composite’s lower absolute strength compared to 40Cr steel, its specific strength performance is marginally superior when density is considered. This finding suggests that the BNNTs/Al composite exhibits both high tensile strength and reduced material density, thereby demonstrating considerable potential for lightweighting applications.

In addition, a comparison Table 1 summarizing density, yield strength, elongation, and specific strength has been added to provide a clearer overview of the mechanical performance of the two materials.

**Figure 10:**
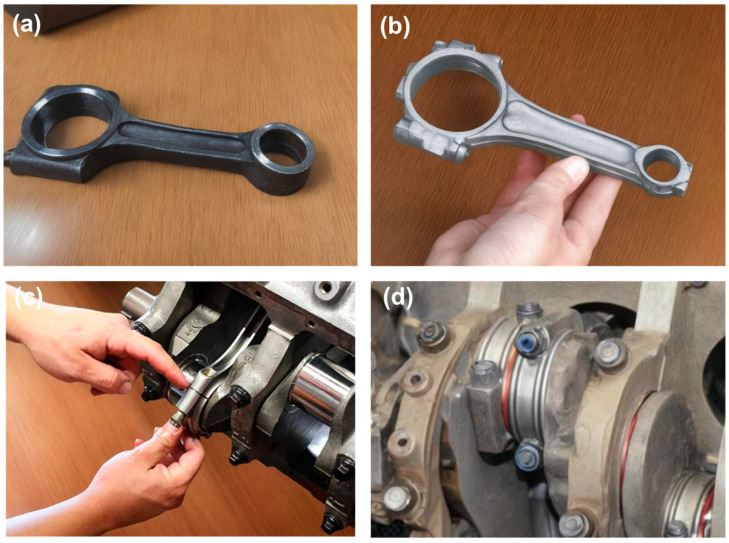
(**a**) shows a conventional 40Cr steel connecting rod as the reference material for comparison; (**b**) presents the innovative BNNTs/Al composite connecting rod developed in this study (**c**) displays a conventional 40Cr steel connecting rod removed from a Toyota Carina model engine (**d**) presents a comparison image where the traditional 40Cr steel connecting rod is replaced with the BNNTs/Al composite automotive connecting rod, visually demonstrating the practical effects of material substitution.

In order to comprehensively evaluate the potential of BNNTs/Al composites in automotive connecting rod applications, this study selected conventional 40Cr steel as the reference material. As illustrated in Figure 10a, the surface morphology of a 40Cr steel connecting rod features mature production processes and reliable performance, thus making it a widely adopted component in the automotive industry. However, the high density of 40Cr steel (7.85 g/cm^3^) poses challenges in the context of the prevailing trend of automotive lightweighting. BNNTs/Al composites, with their low density (2.7 g/cm^3^) and excellent specific strength, hold considerable promise as a novel connecting rod material to replace 40Cr steel. As illustrated in Figure 10b, the physical specimen of the BNNTs/Al composite connecting rod, innovatively fabricated in this study, is presented. It was possible to successfully obtain a composite connecting rod with good surface quality and high dimensional accuracy by means of optimized stir casting and precision forming processes. This outcome unequivocally substantiates the immense potential of BNNTs/Al composites in the fabrication of intricate components. In order to more accurately simulate real automotive operating conditions, a 40Cr steel connecting rod dismantled from a Toyota Carina engine was selected as a reference, as illustrated in Figure 10c. The characterisation of the morphology and properties of the connecting rod under actual service conditions provides a crucial reference point for the design and preparation of composite connecting rods. Concurrently, the effects of material substitution were visually demonstrated by substituting the connecting rod material—replacing the 40Cr steel connecting rod with a BNNTs/Al composite connecting rod (Figure 10d)—laying the foundation for subsequent performance evaluations.

The automotive performance of BNNTs/Al composite connecting rods, including braking force and fuel consumption characteristics, was evaluated using controlled bench-scale tests. Detailed descriptions of the test setup, measurement procedures, repetition protocol, and data processing methods are provided in the Supplementary Materials (Text S4).

**Figure 11:**
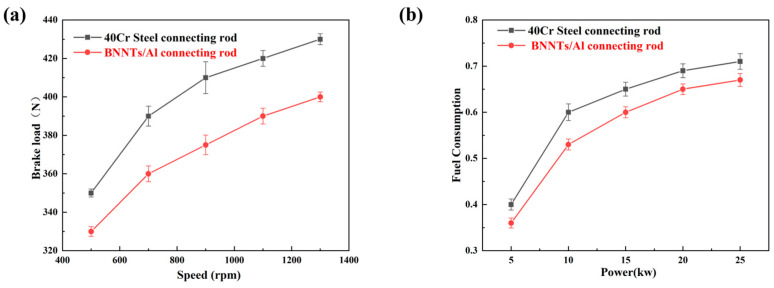
Conventional 40Cr steel connecting rod and BNNTs/Al connecting rod: braking force versus speed (**a**); fuel consumption versus power (**b**).

The braking performance of a vehicle has a direct impact on driving safety and serves as a key indicator for evaluating the reliability of its powertrain system. As a core component in the transmission of engine power, the material and structure of connecting rods have been demonstrated to have a significant influence on braking performance [39]. This study investigates the impact of material lightweighting on automotive braking performance by comparing the braking force of 40Cr steel connecting rods and BNNTs/Al composite connecting rods under different operating conditions. As illustrated in Figure 11a, the braking force variation curves for both connecting rods are presented within the 500–1300 rpm speed range. In general, the BNNTs/Al connecting rod displays a reduced braking force in comparison to its 40Cr steel counterpart, thus indicating an enhanced braking performance. At 1300 rpm, for instance, the 40Cr steel connecting rod generates 428 N of braking force, while the BNNTs/Al connecting rod produces only 400 N—a 6.5% reduction. This disparity is primarily attributable to the smaller moment of inertia and rotational resistance exhibited by the BNNTs/Al connecting rod. On the one hand, the density of BNNTs/Al (2.7 g/cm^3^) is significantly lower than that of 40Cr steel (7.85 g/cm^3^). It has been demonstrated that, at identical dimensions, the lighter mass results in a smaller moment of inertia, thereby facilitating rapid braking. Conversely, the BNNTs/Al composite demonstrates superior specific strength and specific stiffness in comparison to 40Cr steel. When subjected to equivalent loads, the material exhibits reduced deformation and maintains greater stability in the clearance between moving pairs, thereby decreasing rotational resistance. Furthermore, as demonstrated in Figure 11a, the braking force of the connecting rod increases gradually with rising rotational speed, with the increase being less pronounced for the BNNTs/Al connecting rod compared to the 40Cr steel counterpart. This is primarily due to the fact that higher rotational speeds result in greater inertial forces on the connecting rod, which consequently exerts stronger impacts on the braking system. The BNNTs/Al linkage has been demonstrated to be effective in attenuating high-frequency vibrations, thereby ensuring stable braking force output. This effect is attributed to the linkage’s superior vibration damping properties. The exceptional strength, rigidity, and damping properties of BNNTs/Al composite materials effectively mitigate impact loads during emergency braking, enhance braking efficiency, and establish a material foundation for ensuring vehicle and occupant safety.

Fuel economy is a pivotal metric in evaluating the efficiency of automotive powertrains. In the context of dwindling fuel reserves, the enhancement of engine efficiency and the reduction of fuel consumption are of paramount importance for the conservation of resources and the protection of the environment. As a moving component within the engine, the weight and inertial forces of connecting rods directly impact fuel consumption. This section draws parallels between the fuel consumption of 40Cr steel connecting rods and BNNTs/Al connecting rods at varying power levels, thereby elucidating the energy-saving mechanism of material lightweighting. As demonstrated in Figure 11b, the fuel consumption of both materials varies across the 5–25 kW power range. Evidence suggests that vehicles equipped with BNNTs/Al connecting rods exhibit significantly reduced fuel consumption in comparison to those with 40Cr steel connecting rods, thereby demonstrating a substantial reduction in energy expenditure. For instance, at 20 kW, under WLTC-equivalent operating conditions, the BNNTs/Al composite connecting rod exhibits approximately 5.8% lower fuel consumption than the conventional 40Cr steel connecting rod. The low fuel consumption of the BNNTs/Al connecting rod is primarily attributable to its superior specific strength and specific modulus. In the context of engine operation, the connecting rod is subjected to repetitive compressive stresses, with the inertia moment exerting a substantial influence on fuel consumption. BNNTs/Al possesses a density that is one-third that of 40Cr steel, thereby significantly reducing the weight of connecting rods while maintaining strength and stiffness. This reduction in weight decreases the inertia moment, thereby lowering the engine’s power loss. Furthermore, BNNTs provide multiscale reinforcement to the aluminium alloy, thereby enhancing the material’s adaptability to alternating loads. This enhancement contributes to enhanced combustion stability and thermal efficiency in the engine. It is noteworthy that as the power output increases, the discrepancy in fuel consumption between BNNTs/Al and 40Cr steel connecting rods becomes increasingly pronounced. This finding suggests that the energy-saving potential of the system is significant. This finding suggests that the energy-saving benefits of BNNTs/Al composites are more pronounced under high-power operating conditions. It has been demonstrated that, under conditions of elevated velocity and substantial load, there is a substantial augmentation in the inertial forces exerted by the connecting rod. The low density and high strength of BNNTs/Al material ensure effective reduction of vibration and impact, thus ensuring fuel economy.

The enhanced fuel economy not only contributes to a reduction in daily operating costs for vehicle owners but also signifies a substantial advancement in the implementation of energy conservation, emission reduction, and green development principles. To illustrate this point, consider the Toyota Carina model. Following the adoption of BNNTs/Al connecting rods, a 6.3% decrease in Worldwide Harmonized Light Vehicles Test Cycle (WLTC) was observed in comparison to 40Cr steel connecting rods. Concurrently, there was a 5.8% reduction in carbon emissions per 100 km. The implementation of this technology across the entire model range has the potential to achieve significant environmental benefits, including the reduction of fuel consumption by tens of thousands of tons per year and a substantial decrease in carbon dioxide emissions by millions of tons. This contributes to the sustainable development of the automotive industry. The estimated fuel consumption and CO_2_ emission reductions represent potential savings under simplified assumptions, assuming widespread adoption of lightweight BNNTs/Al composite connecting rods and identical driving conditions. The calculations are based on average WLTC fuel consumption data, a simplified vehicle fleet model, and constant annual mileage, without considering variations in driving behavior, vehicle aging, or powertrain configuration.

In summary, BNNT-reinforced aluminium matrix composites, with their outstanding specific strength, specific stiffness, and damping properties, have the potential to significantly enhance the braking safety and fuel efficiency of automotive connecting rods. This provides new insights and material solutions for energy conservation, emission reduction, and lightweight design in automotive powertrain systems, driving the innovative development of green manufacturing technologies. However, the large-scale application of BNNTs/Al composites remains contingent on intensive research in cost control, process optimisation, and mass production. It is inevitable that, in the future, the collaborative innovation of industry, academia, research institutions, and end-users will accelerate the pace of BNNTs’ automotive applications. This will contribute to the development of a green, efficient, and sustainable automotive industrial system, by providing wisdom and strength.

In this study, fatigue testing (such as S–N curve measurement) was not conducted because the primary objective was to establish the feasibility of BNNT-reinforced aluminium composites for connecting rod applications and to evaluate their static mechanical behaviour. Future work will focus on fatigue performance, including high-cycle and low-cycle fatigue tests, to more comprehensively assess the long-term service reliability of BNNTs/Al components. Mechanical properties at elevated temperatures above room temperature were not included in the present investigation. The current work concentrated on the interfacial behaviour, wetting characteristics, and static mechanical properties as a preliminary assessment. In future studies, tensile and compressive tests at temperatures closer to practical engine operating conditions (150–350 °C) will be conducted to further validate the thermal-mechanical stability of BNNTs/Al composites during real service.

## 5. Conclusions

The present study systematically investigates the preparation process, interfacial characteristics, and application of BNNT-reinforced aluminium matrix composites in automotive connecting rods. Through the optimisation of the stirred casting method, the uniform dispersion of BNNTs within the Al matrix and tight interfacial bonding were successfully achieved. Microstructural analysis revealed the presence of BNNTs, which were distributed as either single entities or small clusters without evidence of agglomeration. These nanotubes formed semi-coherent structures at the interfaces (for example, Al/AlN and Al/AlB_2_) that significantly enhanced the efficiency of load transfer. Experiments conducted at elevated temperatures indicated that the optimal level of wettability is achieved at 675 °C, which is characterised by a reduction in the contact angle to 0°. This is accompanied by interfacial reactions that generate AlN and AlB2 phases, which in turn enhance thermal stability and mechanical properties. In addition, BNNTs/Al composite connecting rods demonstrate superior performance in specific strength (compressive specific strength: 142.6 MPa·cm^3^/g vs. 91.3 MPa·cm^3^/g), braking performance (6.5% reduction in braking force), and fuel economy (5.8% reduction in fuel consumption), thereby achieving a balance between lightweighting and high efficiency. Through interface optimisation and process control, BNNTs/Al composites achieve a high strength, low density, and excellent thermal stability, thus offering a potentially viable solution for reducing automotive connecting rod weight and enhancing energy efficiency.

## Figures and Tables

**Table 1:** Comparison of density and mechanical properties of BNNTs/Al composite and 40Cr steel.

Property	BNNTs/Al Composite	40Cr Steel
Density (g/cm^3^)	2.7	7.85
Yield strength—tensile (MPa)	212	590
Elongation (%)	11.4	11.2
Compressive yield strength (MPa)	385	717
Specific strength (MPa·cm^3^/g)	78.5 (tensile)/142.6 (compressive)	75.2 (tensile)/91.3 (compressive)
Elastic modulus (GPa)	70	210

## Data Availability

The original contributions presented in this study are included in the article/Supplementary Material. Further inquiries can be directed to the corresponding author.

## Supplementary Materials

The following supporting information can be downloaded at: https://www.mdpi.com/article/10.3390/ma19010048/s1, Figure S1: Morphology of pristine BNNTs: (a) SEM image showing an interconnected network of BNNTs with high aspect ratios; (b) TEM image revealing the hollow and multi-walled tubular structure of individual BNNTs; Figure S2: Process Flow for Fabricating Automotive Connecting Rods from Boron Nitride/Aluminum Composite Materials (a) Dimensioning; (b) Mold Machining; (c) Connecting Rod Blank; (d) Connecting Rod Finishing; Figure S3: Experimental Setup for High-Temperature, High-Vacuum Contact Angle Measurement; Figure S4: Dimensions of Mechanical Property Test Specimens; Figure S5:Raman spectra of pristine BNNTs and BNNTs after composite processing; Text S1: Process for Manufacturing Automotive Connecting Rods from BNNTs/Al Composite Materials; Text S2: Contact angle testing; Text S3: Mechanical Properties Testing; Text S4: Automotive Performance Testing Methodology.
