# Supplementary material for: Preparation of Boron Nitride Nanotube/Aluminum Matrix Composites and Their Application in Automotive Connecting Rods
Source: Materials (Basel). 2025 Dec 22;19(1):48. doi: 10.3390/ma19010048 (PMC12786862; doi:10.3390/ma19010048)
Supplement: Supplementary file 1 [file materials-19-00048-s001.zip › materials-4045410-supplementary.pdf]

Preparation of Boron Nitride Nanotube/Aluminum Matrix Composites and Their Application in Automotive Connecting Rods

Yong HUANG, Bingzhan ZHANG*, Han ZHAO, Qingtao LI, Jianbo BI

Hefei University of Technology, School of Automotive and Traffic Engineering, Hefei, 230009, China

E-mail: mailto:zhangbingzhan@hfut.edu.cn

TEXT S1

Process for Manufacturing Automotive Connecting Rods from BNNTs/Al Composite Materials

This study uses the stirred casting method to make BNNTS/Al composite connecting rods. Optimising the process parameters achieves uniform dispersion of the BNNTS within the Al matrix and strong interfacial bonding, thereby enhancing the mechanical properties and operational reliability of the composite material. Based on extensive experimental research and theoretical analysis, a comprehensive fabrication process for BNNTS/Al composite connecting rods has been developed, paving the way for their industrial application. The process for manufacturing automotive connecting rods from BNNTS/Al composites comprises the following steps:

(a) Dimensioning: Using computer-aided design (CAD) software such as CATIA or UG to optimise the geometric dimensions and structural parameters of the connecting rod based on engine specifications and performance requirements. Finite element analysis (FEA) simulates stress distribution and deformation behaviour under actual working loads, enabling fatigue life prediction and ensuring dimensional and structural compliance. At the same time, adjustments are made to accommodate the material's casting characteristics, including pouring system design and solidification shrinkage compensation, to guarantee casting quality.

(b) Mold machining: Based on the optimised dimensions and structure of the connecting rod, advanced manufacturing techniques such as CNC machining centres or EDM are used to make the casting moulds. Mould materials are usually made of high-strength, wear-resistant hot-work tool steels such as H13 or 8407. Mould surfaces undergo polishing and coating treatments to improve the quality of the cast surface and demoulding performance. To boost production efficiency, multi-cavity mould designs are commonly adopted for the batch production of connecting rods. Additionally, auxiliary structures such as gating systems, risers and vent channels are integrated into the moulds to ensure the quality and dimensional accuracy of the castings.

(c) Connecting rod blank preparation: Use the stirred casting process to make BNNTS/Al composite connecting rod blanks. First, melt the aluminium alloy and introduce the BNNTS particles. Use mechanical stirring to achieve uniform dispersion of the particles within the molten aluminium. During stirring, strictly control the speed, duration and design of the agitator to prevent agglomeration and precipitation of the BNNTS particles. The melt temperature and holding time must also be regulated to ensure optimal interface bonding between the BNNTS particles and the aluminium matrix. After stirring, pour the BNNTS/Al composite melt into a preheated connecting rod mould. Vibration and pressure are then applied to facilitate filling and shaping of the melt. Once cooled and solidified, the connecting rod blank is formed.

(d) Connecting rod finishing: The prepared BNNTS/Al composite connecting rod blanks are finished to meet their application requirements. First, non-destructive testing methods such as X-ray inspection and ultrasonic testing are employed to inspect the connecting rod blanks and eliminate those with internal defects. Qualified connecting rod blanks then undergo a series of finishing operations, including trimming, drilling, milling and polishing, using CNC machining centres and specialised fixtures to achieve the dimensional accuracy and surface quality specified in the drawings. Appropriate cutting parameters and tools must be selected during finishing to prevent BNNTS particle detachment and matrix pull-out. Finally, the finished connecting rods undergo mechanical property and bench testing to evaluate critical performance indicators such as fatigue strength and vibration characteristics.

TEXT S2

Contact angle testing:

This experiment explored the wetting properties of BNNT/Al composite systems under varying temperature conditions, employing a series of rigorous and systematic operational steps. First, A380 aluminium alloy was precision-cut into standardised cubic substrates using a wire cutter. Next, a stepwise sanding technique combined with chemical etching using a 0.1 mol/L HCl solution effectively removed the oxide film from the aluminium surface, minimising potential interference with contact angle measurements. The treated substrates were then stored in kerosene to prevent re-oxidation. Dry powder hydraulic forming technology was then used to compress BNNT powder into uniform cylindrical carbon substrates under a pressure of 400 MPa and for a holding time of 90 seconds. These substrates served as the support base for the composite system. The core measurement phase then commenced using a high-temperature, high-vacuum contact angle analyser (OCA25-HTV1800). Under strictly controlled vacuum conditions ($<1\times10^{-5}$ Pa), an appropriate volume of test liquid was dispensed onto the sample surface via the sessile drop method. A series of temperature observation points were selected (298 K, 933 K, 1033 K and 1133 K). The temperature was gradually increased to each observation point at a rate of 5 K/min, followed by a holding period to ensure thermal equilibrium. At each temperature, the droplet morphology was recorded and photographed precisely. Contact angles on both sides of the droplet were measured and calculated, and the average value was taken to enhance data accuracy and reliability.

TEXT S3

Mechanical Properties Testing:

This experiment aims to evaluate the mechanical properties of aluminium-based composites at room temperature and elevated temperatures. This will provide a deeper understanding of how they respond mechanically to different environmental conditions. To this end, the UTM5105 universal testing machine, manufactured by Zhuhai Nankai Electric Equipment Co., Ltd., has been selected as the primary testing apparatus. This equipment features high precision, a wide load capacity range and a stable control system, and is capable of meeting the demands of complex mechanical property testing of materials. Regarding the experimental design, the room-temperature mechanical property testing was conducted under standard laboratory conditions, with the temperature controlled to 20°C ± 2°C and the relative humidity maintained to 50% ± 10%. These conditions simulate a typical working environment for evaluating material performance.

High-temperature mechanical property testing is designed to be conducted in a furnace chamber at high temperatures, with a protective atmosphere of high-purity argon gas. This prevents material oxidation at high temperatures, ensuring accurate test results. The flow of argon gas is precisely regulated to maintain a stable inert environment within the furnace chamber. Test temperatures are set according to the requirements of the experiment and are maintained until its conclusion. Regarding specimen preparation and testing methods, tensile specimen dimensions are designed in accordance with ASTM E8 or relevant international standards, ensuring representativeness and compliance with testing requirements. Tensile specimen dimensions are shown in Figures 2–4. The tensile speed is precisely controlled at 0.5 mm/min to balance testing efficiency with data accuracy. Five tensile bars were randomly selected from each sample for testing to minimise random errors. After testing, statistical methods were used to calculate the mean and standard deviation of the five test results, providing a more accurate representation of the material's mechanical properties. The experimental procedures encompassed specimen preparation, equipment calibration, ambient temperature testing and elevated temperature testing. Specimen preparation involved machining aluminium matrix composite tensile specimens to standard dimensions and performing the necessary surface treatments to mitigate the influence of surface defects on the results of the tests.

Calibrating the equipment ensures testing accuracy. Room-temperature testing involves conducting tensile tests on specimens under standard environmental conditions and recording the results. Preparing the high-temperature furnace involves preheating the furnace chamber to the set temperature and introducing an argon protective atmosphere. Once the temperature has stabilised, the specimens are transferred quickly into the furnace chamber. High-temperature testing involves tensile testing of specimens at a rate of 0.5 mm/min under the protective atmosphere, with the relevant data being recorded. Data processing and analysis involves statistically analysing room-temperature and high-temperature test data and calculating statistical measures such as the mean and standard deviation. Comparative analysis explores the influence of temperature on the mechanical properties of aluminium-based composites. This experimental design and procedure enables the study to comprehensively and accurately evaluate the mechanical properties of aluminium-based composites under both room temperature and high temperature conditions, providing reliable data to support further material applications.

TEXT S4

Automotive Performance Testing Methodology

To evaluate the influence of BNNTs/Al composite connecting rods on automotive performance, braking force and fuel consumption tests were conducted under controlled bench-scale conditions. All tests were designed to ensure that the connecting rod material was the only variable, while engine configuration, operating parameters, and boundary conditions were kept identical.

Braking Force versus Speed Test

The braking force versus speed characteristics were obtained using an engine dynamometer–based bench testing system. The engine was operated over a rotational speed range of 500–1300 rpm under controlled braking conditions. Braking torque was continuously measured by the dynamometer and subsequently converted into braking force using standard mechanical relationships.

For each connecting rod material (BNNTs/Al composite and conventional 40Cr steel), three independent tests were performed at each speed point to ensure repeatability. The reported braking force values represent the average of the repeated measurements, and the corresponding standard deviations were used to quantify experimental scatter.

Fuel Consumption versus Power Test

Fuel consumption as a function of engine output power was evaluated under steady-state operating conditions using the same engine dynamometer setup. The engine was stabilized at discrete power levels ranging from 5 to 25 kW. Fuel consumption was measured using a calibrated fuel mass flow measurement system, and the instantaneous fuel consumption rate was recorded once thermal and mechanical steady state was achieved. Each operating condition was repeated three times for both connecting rod materials. The mean values were reported, and the experimental scatter was quantified using standard deviation analysis. These data were used to construct the fuel consumption versus power curves.

WLTC Fuel Consumption and CO_2 Emission Estimation

The Worldwide Harmonized Light Vehicles Test Cycle (WLTC) fuel consumption and CO_2 emission reductions were not measured directly on a full vehicle. Instead, they were estimated based on the experimentally measured fuel consumption data obtained from the bench tests. The measured fuel consumption rates were combined with standard WLTC driving cycle weighting factors to estimate the corresponding WLTC fuel consumption and CO_2 emissions. This approach allows a consistent comparison of the potential energy-saving and emission-reduction effects associated with the lightweight BNNTs/Al composite connecting rod, while avoiding the uncertainty introduced by additional vehicle-level variables.

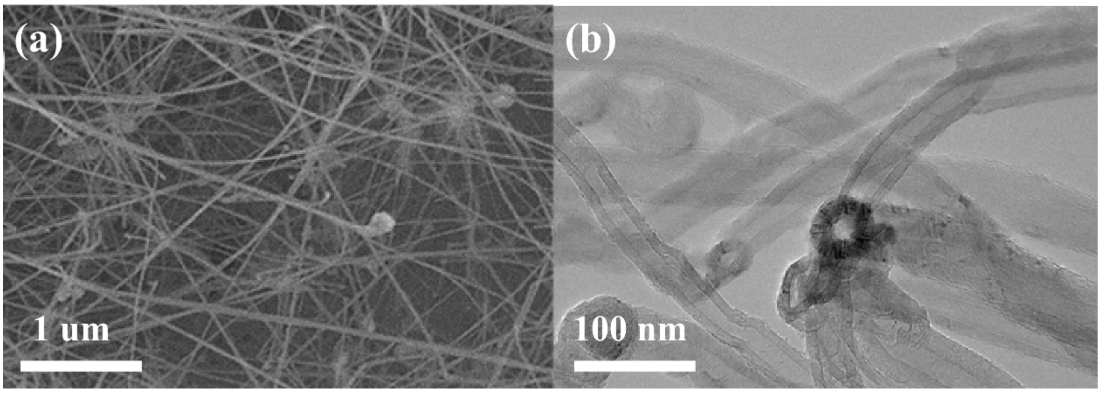

Figure S1. Morphology of pristine BNNTs: (a) SEM image showing an interconnected network of BNNTs with high aspect ratios; (b) TEM image revealing the hollow and multi-walled tubular structure of individual BNNTs.

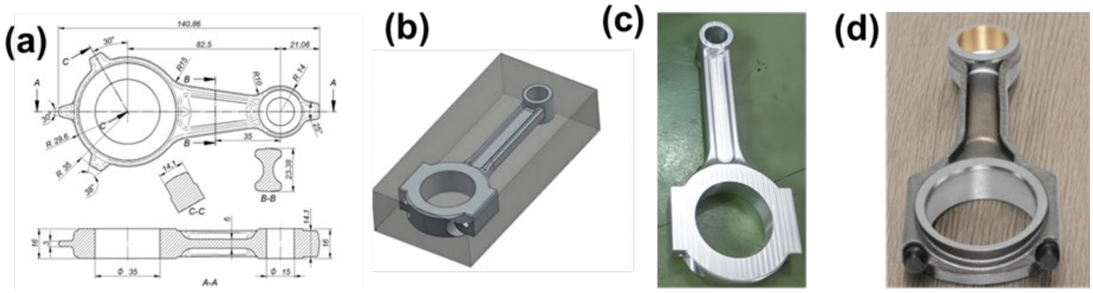

Figure S2. Process Flow for Fabricating Automotive Connecting Rods from Boron Nitride/Aluminum Composite Materials (a) Dimensioning; (b) Mold Machining; (c) Connecting Rod Blank; (d) Connecting Rod Finishing

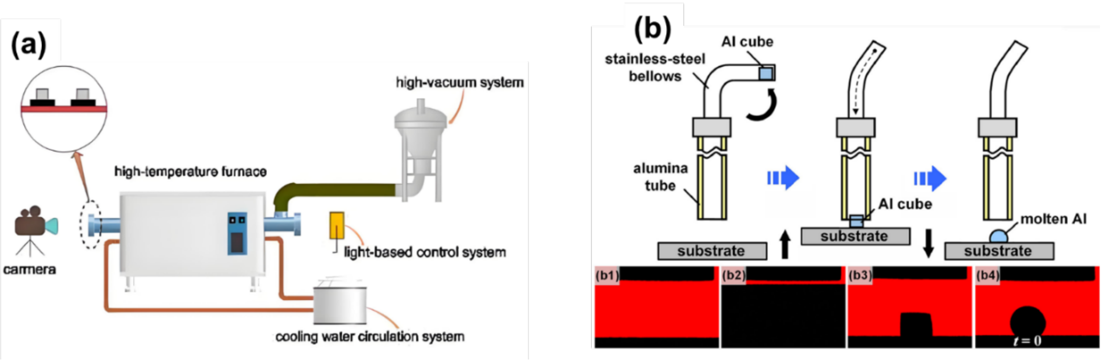

Figure S3. Experimental Setup for High-Temperature, High-Vacuum Contact Angle Measurement

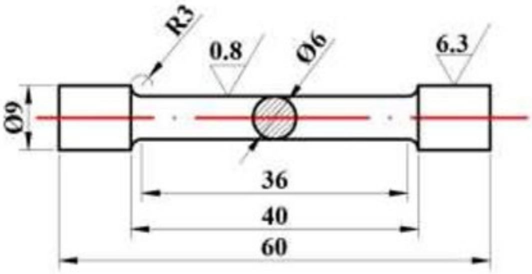

Figure S4: Dimensions of Mechanical Property Test Specimens

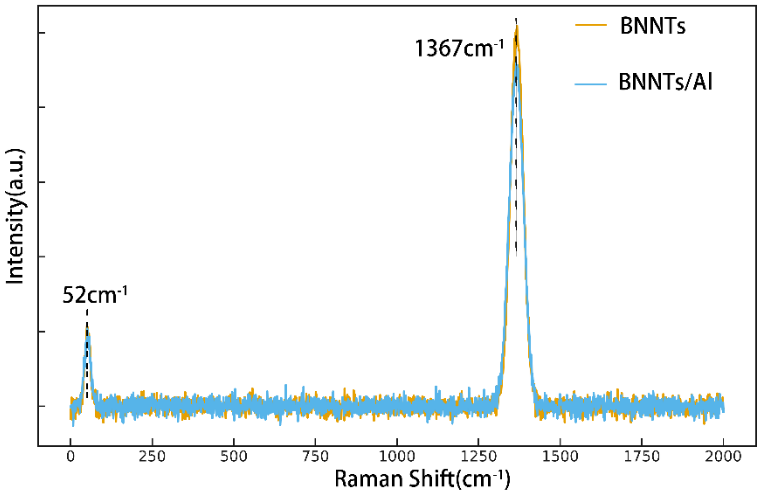

Figure S5. Raman spectra of pristine BNNTs and BNNTs after composite processing.
